# Emotional Support, Emotional Exhaustion, Mental Health, and Turnover Intention Among China Nurses in Emergency Departments: A Cross-Sectional Survey

**DOI:** 10.1097/jnr.0000000000000710

**Published:** 2025-11-06

**Authors:** Jing Li, Qiuxiang Zhang, Yinglong Duan, Mingyue Huang, Meiying Guo

**Affiliations:** 1 Nursing Department, The Third Xiangya Hospital, Central South University, Changsha, Hunan, China; 2 Xiangya School of Nursing, Central South University, Changsha, China; 3 Emergency Department, The Third Xiangya Hospital, Central South University, Changsha, Hunan, China; † Contributed equally

**Keywords:** emergency department nurses, turnover intention, emotional exhaustion, emotional support, conservation of resources theory

## Abstract

**Backgrounds:**

In China, emergency department (ED) nurses frequently face high turnover rates and emotional exhaustion, phenomena that are intensified by the demanding nature of their roles. While emotional exhaustion significantly influences nurses intention to leave, the potential mitigating effects of factors such as emotional support have not been thoroughly explored.

**Purpose:**

This study was designed to investigate the mediating role of emotional support between emotional exhaustion and turnover intention among ED nurses in China and to examine the factors of influence on turnover intention.

**Methods:**

A cross-sectional research design was used to collect data from 427 ED nurses from different hospitals across China using convenience sampling. The research instruments used included the Emotional Support Scale, Emotional Exhaustion Scale, Self-Rated Mental Health Status Scale, and Turnover Intention Scale.

**Results:**

The findings showed not proactively having chosen a career in nursing (β=0.096, *p*<.05), greater emotional exhaustion (β=0.318, *p*<.001), satisfactory economic status (β=−0.196, *p*<.001), better physical condition (β=−0.128, *p*<.05), and more emotional support (β=−0.092, *p*=.032) to correlate negatively with turnover intention and emotional support to mediate the relationship between emotional exhaustion and intention to leave (β=−0.095, Bootstrap 95% CI [−0.152, −0.046], *p*<.001), accounting, collectively, for 34.8% of the total effect (β=−0.273, Bootstrap 95% CI [−0.364, −0.175], *p*=.002).

**Conclusions:**

Enhancing emotional support reduces turnover intention by mitigating the effects of emotional exhaustion on ED nurses in China. Targeted interventions that bolster emotional support systems, together with strategies that address financial satisfaction and physical well-being, will be essential to enhancing both nursing staff retention and overall patient care quality in China’s demanding health care environment.

## Introduction

Over the past few years, the nursing profession has faced unparalleled challenges and pressures globally. The International Council of Nurses (ICN) has underscored that between 40% and 80% of nurses worldwide have experienced symptoms of psychological stress (ICN, 2023). Moreover, the intent of nurses to resign from their positions has surged to 20% or more, leading to an annual turnover rate of hospital staff surpassing 10% (ICN, 2023). These figures illustrate the intense pressures faced by nursing professionals, particularly in high-stress environments like emergency departments (EDs). ED nurses, in particular, experience significantly higher turnover rates compared with nurses in other departments, primarily due to the demanding work environment, exposure to critical situations, and heightened work-related stress (Jiang et al., 2023). The high turnover of ED nurses not only increases recruitment and training costs for hospitals but also disrupts patient care, as these nurses play a crucial role in the immediate, life-saving care of critically ill patients (Wubetie et al., 2020).

In China, the pressure on ED nurses is particularly acute due to the growing demand for health care services, driven by both social and demographic factors (Jiang et al., 2023). ED nurses in China face an exceptionally high patient load, intense work conditions, and the complexities associated with providing care in an environment characterized by frequent medical emergencies (Jiang et al., 2017). Moreover, the unique challenges within China’s health care system, such as uneven distribution of medical resources, large patient volumes, and a rapidly aging population, further compound the stress faced by ED nurses (Chen et al., 2021; Jakovljevic et al., 2023). These factors, combined with cultural expectations that place additional emotional and physical demands on health care workers, contribute to a highly stressful working environment. As a result, ED nurses are particularly vulnerable to emotional exhaustion and burnout, which may increase turnover intention and negatively impact mental health (Norvell et al., 2023; Song et al., 2023).

Conservation of Resources Theory (CART) provides a robust theoretical framework for understanding emotional exhaustion and turnover intention in nurses. According to CART, individuals are motivated to conserve resources such as energy, time, and personal capabilities, while stressors, particularly work-related stress, deplete these resources, leading to negative outcomes such as job dissatisfaction, burnout, and turnover intention (Liu et al., 2020a). ED nurses must not only cope with daily intense work pressures but also face strong emotional demands from patients and their families (Jiang et al., 2023). Specifically, the high emotional and physical demands placed on ED nurses deplete their psychological resources, exacerbating emotional exhaustion, which is a key predictor of turnover intention, defined as the desire or intent to leave one’s job (Jiang et al., 2023). Turnover intention refers to the likelihood of an employee considering leaving their current position, and is often triggered by emotional exhaustion and reduced job satisfaction (Hu et al., 2022). In this context, emotional support can be conceptualized as a crucial resource that buffers the negative effects of emotional exhaustion on turnover intention. Emotional support from colleagues, supervisors, and health care organizations helps replenish the depleted psychological resources of ED nurses, thereby mitigating the impact of emotional exhaustion on intention to leave (Green et al., 2022). Mental health refers in this study to an individual’s overall perception of their psychological well-being. This self-assessed measure reflects the subjective evaluation of nurses of their ability to manage stress, maintain emotional balance, and function effectively in both personal and professional roles (Alonazi et al., 2023). In high-pressure environments such as EDs, self-assessment tools provide valuable insights into the relationship between emotional exhaustion and turnover intention (Alonazi et al., 2023). In providing adequate emotional support, health care organizations can foster resilience among nurses, reducing emotional exhaustion and turnover intention (Maddock, 2024).

The central question addressed in this study is: To what extent does emotional support mediate the relationship between emotional exhaustion and turnover intention among ED nurses in China? In this study, CART is integrated with emotional support theory to examine how emotional support functions as a resource that mitigates the negative effects of emotional exhaustion. Specifically, the potential of emotional support to reduce turnover intention, which is critical for the retention of ED nurses and their mental well-being, is assessed. The theoretical framework informs the following hypotheses: (a) emotional exhaustion is positively correlated with turnover intention, (b) emotional support is negatively correlated with turnover intention, and (c) emotional support mediates the relationship between emotional exhaustion and turnover intention. In this study, a gap in the existing literature is addressed by exploring these relationships within the specific context of China’s health care system and cultural environment, and actionable insights are provided for health care administrators and policymakers to enhance the psychological well-being and retention of ED nurses. By emphasizing the importance of emotional support systems, the researchers aim to contribute to the development of strategies that improve nurse retention and ensure quality of care in high-pressure emergency settings.

## Methods

### Design and Ethical Approval

This cross-sectional study received approval from the Ethics Committee of the IRB at The Third Xiangya Hospital of Central South University (approval – quick│22297) and from the ethics committee of each participating hospital.

### Research Sample

A convenience sampling method was used, and nurses were selected from multiple hospitals in Hunan Province between December 2021 and January 2022. To ensure diversity in work environments, nurses were recruited from hospitals of different levels within China’s health care system, including four tertiary hospitals, 10 secondary hospitals, and 3 primary hospitals. Tertiary hospitals are large medical centers that provide specialized and advanced care and often serve as regional or national referral centers; secondary hospitals offer general medical services with some specialized care, typically at the city or county level; and primary hospitals serve local communities through providing basic health care services. Including ED nurses from these different hospital levels helps facilitate a more comprehensive understanding of how different hospital environments, resources, and workloads may influence emotional exhaustion and turnover intention among nurses in general. This diversity is expected to enhance the representativeness of the findings across varied health care settings.

Inclusion criteria used during participant recruitment were the following: (a) registered nurse with a valid nursing license, (b) currently working as a clinical nurse in a hospital ED, and (c) volunteer to participate in the study. Exclusion criteria included: (a) nurses with less than 6 months of experience in an ED and (b) nurses not currently working in the ED due to pregnancy, breastfeeding, or other reasons. The questionnaire was distributed via the Wenjuanxing platform, a widely used online survey tool in China. The purpose and significance of this study were explained to the prospective participants electronically via the survey platform, and informed consent was obtained. The participants were concurrently introduced to the purpose, content, and requirements of the survey, which could be completed at each participant’s convenience by accessing the provided link. The survey was anonymous, and all responses were automatically collected by the platform to ensure confidentiality. To enhance data quality, the survey was designed to include validation checks, such as required responses for key questions and randomization of question order, to reduce response bias. In addition, a follow-up reminder was sent to participants after 1 week to encourage completion. After data collection, the responses were reviewed for completeness, and any incomplete questionnaires were excluded from the analysis.

A total of 460 questionnaires were distributed, and 451 were collected for a recovery rate of 98.04%. Twenty-four questionnaires with incomplete information were excluded, leaving 427 valid questionnaires, giving an efficiency rate of 94.68%. To determine the minimum required sample size, Structural Equation Modeling (SEM) was used for the path analysis and the general rule of thumb suggesting a minimum of 10 samples per estimated parameter was followed. Based on the approximately 30 parameters in the model, the required minimum sample size was calculated as 300. Thus, the 427 valid samples included in this study were sufficient to ensure statistical power and model robustness.

### Research Methodology

#### Research tools

1. Demographics Datasheet: Demographic data collected included gender, age, educational level, single status, employment, years of service, title, position, hospital level, economic status, physical condition, and how they chose to pursue nursing. Economic status, a self-rated item that reflects respondent satisfaction with their financial situation, is a variable that has been linked to job satisfaction and retention in previous studies. Physical condition refers to self-rated physical health, indicating the nurse’s overall physical ability to perform their duties and manage the demands of their work.
2. Emotional Support Scale: This scale was developed by Methot et al. (2016) in 2015 based on the research scale of Schaefer et al. (1981) and Mossholder et al. (2005): (1) “My coworkers share related personal experiences as an alternative perspective to my problems.” (2) “My coworkers provide encouragement and emotional support.”(3) “My coworkers boost my spirits when I feel low.” (4) “My coworkers listen to me when I’m frustrated about something and need to vent.” (5) “My coworkers empathize with my concerns and feelings.” This scale is scored on a 5-point Likert scale, with 1=“*strongly disagree*,” 2=“*disagree*,” 3=“*unsure*,” 4=“*agree*,” and 5=“*strongly agree*.” Scale items are averaged to generate a single emotional support score, with higher scores indicating higher emotional support. The Cronbach’s α coefficient in this study was .912.
3. Emotional Exhaustion Scale: This scale was developed by Boswell et al. (2015) based on the Maslach Burnout Scale (Watkins et al., 2015). Scale items include (1) “I feel emotionally drained from my work”; (2) “I feel burned out from my work”; and (3) “I feel exhausted when I think about having to face another day on the job.” This scale is scored using a 7-point Likert scale, with 1=“*strongly disagree*,” 2=“*disagree*,” 3=“*somewhat disagree*,” 4=“*not too sure*,” 5=“*somewhat agree*,” 6=“*agree*,” and 7=“*strongly agree*.” Item scores are averaged to provide a single emotional exhaustion score, with higher scores indicating higher levels of emotional exhaustion. Cronbach’s α coefficient of the Emotional Exhaustion Scale in this study was .944.
4. Turnover Intention Scale: This 6-item scale was developed by Michaels and Spector (1982), with items 1 and 6 addressing turnover intention I (i.e., likelihood of quitting your current job), items 2 and 3 addressing turnover intention II (i.e., motivation to find another job), and items 4 and 5 addressing turnover intention III (i.e., likelihood of seeking another job). This scale is scored on a 4-point Likert scale, with 4=“*often*,” 3=“*occasionally*,” 2=“*rarely*,” and 1=“*never*” and higher total scale scores indicating stronger willingness to leave. The indicator value is the ratio of the actual score to the ideal maximum score, with a total mean score of ≤1 indicating very low, >1 to ≤2 indicating low, >2 to ≤3 indicating high, and >3 indicating very high turnover intention. The Cronbach’s α coefficient of the Chinese version scale was .814 in this study.
5. Self-rated Mental Health Scale: This single-item scale was developed by Mawani and Gilmour (2010), with respondents asked to answer the question, “How is your general health?”. A 5-point Likert scale is used, with 1=“*very poor*,” 2=“*poor*,” 3=“*fair*,” 4=“*good*,” and 5=“*very good*.” Scores ≥4 are interpreted as a positive self-assessment of health and ≤3 as a negative self-assessment of health. The Cronbach’s α value for this single-item scale is not applicable, but its use in numerous large-scale surveys demonstrates its validity as a global indicator of mental health.

### Statistical Methods

Descriptive statistical analyses, including the calculation of means, frequencies, component ratios, and standard deviations, were initially conducted to provide a basic description of the variables, which include emotional support, emotional exhaustion, mental health, and turnover intention. *t* tests and analysis of variance (ANOVA) were employed to identify factors potentially influencing turnover intention. For the ANOVA, post hoc comparisons using Tukey’s HSD test and Games-Howell test were conducted to determine specific group differences. Subsequently, Pearson correlation analysis was utilized to assess the degree of association between emotional support, emotional exhaustion, mental health, and turnover intention.

To address potential multicollinearity among the variables, the variance inflation factor was computed, with the results indicating no significant multicollinearity concerns. Based on these initial findings, further multiple stepwise regression analyses (α-input=.10, α-output=.15) were performed to identify the most significant factors influencing turnover intention. To more thoroughly examine the relationships between emotional support and, respectively, emotional exhaustion and turnover intention, structural equation modeling (SEM) was employed to explore the potential mediating effects of emotional support. SEM is an advanced statistical method that enables the simultaneous analysis of multiple variables and their direct and indirect effects, making it particularly suitable for testing complex relationships and mediation models. Given the presence of multiple mediation pathways in our conceptual model, SEM allowed for a more comprehensive assessment. In addition, bootstrap analysis was conducted to validate the path coefficients and test the robustness of the mediation effects. This resampling technique is particularly useful in providing confidence intervals for path coefficients, ensuring that the estimated effects are statistically significant and reliable. All variables were standardized to z-scores to ensure consistency across different scales and improve model interpretability. A significant level of .05 (two-sided test) was applied to all of the statistical tests.

## Results

### Sociodemographic Characteristics

The sociodemographic characteristics of the 427 participants, with ages ranging from 19 to 55 (30.9±6.12) years, are presented in Table 1 below. Most (87.8%; *n*=375) were females, 78.2% held university diplomas, and 74.7% were married. Average work experience was 8.07 years, with most (82.2%, *n*=351) working for over 5 years. The majority (71.9%) had chosen to major in nursing, 96.2% held professional titles (e.g., charge nurse), 94.4% were clinical nurses, and most (76.1%) worked at tertiary hospitals.

**Table 1:** Univariate Analysis Results for Turnover Intention​​​​ (N = 427)

Item	*n*	Constituent Ratio (%)	Total Score of Turnover Intention	*F/t*	*p*
			*M*±*SD*		
Gender				1.172	.242
Male	52	12.2	15.15±3.52		
Female	375	87.8	14.51±3.71		
Age (y)				7.179	<.001*
<25	59	13.8	13.71±3.92 ^a, b^		
25–29	135	31.6	15.16±3.46 ^a^		
30–39	192	45.0	14.91±3.60 ^a^		
≥40	41	9.6	12.54±3.69 ^b^		
Educational level				2.593	.052
Technical secondary school	2	0.5	17.50±0.71		
Junior college	67	15.7	13.59±3.67		
Bachelor’s degree	334	78.2	14.72±3.67		
Master’s degree or higher	24	5.6	15.42±3.66		
Chose nursing specialty independently				−5.911	<.001*
Yes	307	71.9	13.96±3.69		
No	120	28.1	16.22±3.16		
Single				1.786	.075
Yes	108	25.3	15.14±3.50		
No	319	74.7	14.41±3.74		
Years of work experience				2.778	.041*
<3	31	7.3	13.94±3.79 ^x, y^		
3–4	45	10.5	14.44±3.61 ^x, y^		
5–9	150	35.1	15.28±3.53 ^x^		
≥10	201	47.1	14.59±3.69 ^y^		
Title				2.883	.022*
Nurse	64	15.0	13.97±3.79		
Primary nurse	170	39.8	15.16±3.49		
Nurse in charge	177	41.5	14.46±3.80		
Deputy chief nurse or more	14	3.3	12.79±3.02		
Chief nurse	2	0.5	11.00±2.83		
Position				1.847	.159
Clinical nurse	403	94.4	14.67±3.72		
Head nurse	20	4.7	13.05±2.74		
Department head nurse or above	4	0.9	14.50±3.11		
Hospital Level				0.79	.500
Level I	5	1.2	13.40±2.30		
Level II	94	22.0	14.73±3.59		
Level III	325	76.1	14.54±3.74		
Other	3	0.7	17.22±1.15		
Economic status				24.362	<.001*
Very dissatisfied	30	7.0	17.40±2.82 ^α^		
Somewhat dissatisfied	78	18.3	15.86±3.07 ^α^		
Neutral	196	45.9	15.05±3.39 ^α, β^		
Somewhat satisfied	109	25.5	12.68±3.49 ^β^		
Very satisfied	14	3.3	10.07±3.85 ^γ^		
Physical condition				17.466	<.001*
Very poor health	5	1.2	18.40±2.88 ^a^		
Poor health	52	12.2	16.02±3.18 ^a^		
Fair	192	45.0	15.44±3.17 ^a^		
Good health	146	34.2	13.56±3.72 ^b^		
Very good health	32	7.5	11.28±4.02 ^c^		

*Note.* For the age variable, different superscript letters (a, b) indicate significant differences between groups (*p*<.05); For the years of work experience variable, different superscript letters (x, y) indicate significant differences between groups (*p*<.05); For the economic status variable, different superscript Greek letters (α, β, γ) indicate significant differences between groups (*p*<.05); For the physical condition variable, different superscript letters (a, b, c) indicate significant differences between groups (*p*<.05). Due to the violation of homogeneity of variance, Welch’s ANOVA and Games-Howell post hoc tests were used.

### Univariate Analysis of Turnover Intention of ED Nurses With Different General Information

One-way ANOVA and *t* test results revealed significant differences in turnover intention scores based on age, how the participant chose to pursue nursing, years of work experience, title, economic status, and physical condition. Moreover, the post hoc analysis results showed participants aged 25–29 and 30–39 years had significantly higher turnover intention scores than their peers aged ≥40 years. Also, participants with 5–9 years of work experience had significantly higher turnover intention scores than those with ≥10 years of experience. Although those holding the title of “Primary Nurse” had higher turnover intention scores than those holding more senior titles, e.g., “Nurse in Charge” or “Deputy Chief Nurse,” post hoc test results did not show significant differences among specific title groups. Lower self-rated economic status was associated with higher turnover intention, with those self-rating as “very dissatisfied” or “somewhat dissatisfied” having significantly higher turnover intention scores than those self-rating as “somewhat satisfied” or “very satisfied.” Moreover, turnover intention declined with higher self-rated physical health, with significant differences observed among most health categories (*p*<.05). Participants who reported “very poor health” had the highest mean turnover intention scores, while those in “very good health” had the lowest mean score. Related information is summarized in Table 1.

### Turnover Intention and Dimension Scores

The mean total score for turnover intention was (14.59±3.69), and average scores in each dimension were Ⅰ (4.49±1.61), Ⅱ (4.54±1.60), and Ⅲ (5.56±1.28). Twelve participants (2.8%) had very low intention to leave, 115 (26.9%) had low intention to leave, 262 (61.4%) had high intention to leave, and 38 (8.9%) had very high intention to leave.

### Emotional Support, Emotional Exhaustion, and Mental Health Scores

The mean total scores for emotional support, emotional exhaustion, and mental health scores were 18.81 (±2.74), 12.06 (±4.61), and 3.42 (±0.814), respectively.

### Correlations Between Turnover Intention and Emotional Support, Emotional Exhaustion, and Mental Health

Pearson correlation analysis showed turnover intention to correlate negatively with both emotional support and mental health status and positively with emotional exhaustion (*p*<.05). Refer to Table 2 for correlation coefficients.

**Table 2:** Correlation Between Turnover Intention and, respectively, Emotional Support, Emotional Exhaustion, and Mental Health (N=427)

Item	Turnover Intention
	*r*
Emotional support	−.273*
Emotional exhaustion	.484*
Self-rated mental health	−.394*

* Significantly correlated at the .05 level (two-sided).

### Multiple Regression Analysis of Factors of Influence on Turnover Intention

The multiple stepwise regression analysis identified satisfaction with economic status, physical condition, how they chose to pursue nursing, average emotional exhaustion score, and average emotional support score as predictors of turnover intention, accounting for 33.3% of the variance in turnover intention. See Table 3 for detailed results.

**Table 3:** Multiple Regression Analysis Results for Turnover Intention (N = 427)

Item	*B*	*SE*	β	*t*	95% CI	*p*
Economic Status	−0.783	0.184	−0.196	−4.264	[−1.145, −0.422]	<.001*
Physical Condition	−0.568	0.200	−0.128	−2.832	[−0.962, −0.174]	.005
Chose Nursing Specialty Independently	0.790	0.347	0.096	2.281	[0.109, 1.471]	.023
Emotional Support	−0.620	0.288	−0.092	−2.152	[−1.186, −0.054]	.032
Emotional Exhaustion	0.761	0.108	0.318	7.023	[0.548, 0.974]	<.001*

*Note*. *R*=.577, the adjusted *R* ^2^=.333, *F*=42.093, *p*<.05.

β = standardized coefficient; *B* = unstandardized coefficient; *SE* = standard error.

### Emotional Support as a Mediator Between Emotional Exhaustion and Turnover Intention

Emotional support was found to have a significantly negative direct effect on turnover intention (β=−0.178, Bootstrap 95% CI [−0.255, −0.073], *p*<.001) and to be negatively associated with emotional exhaustion (β=−0.213, *SE*=0.047, CR=−4.495, *p*<.001). Emotional exhaustion was shown to be positively associated with turnover intention (β=0.446, *SE*=0.043, CR=10.491, *p*<.001).

**Figure 1:**
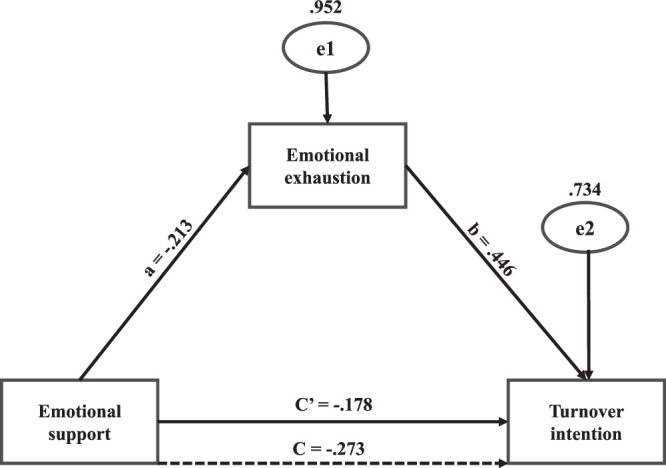
Mediating Role of Emotional Support on the Relationship Between Emotional Exhaustion and Turnover Intention

The results of the mediation analysis, validated through bootstrap resampling, show emotional support to influence turnover intention indirectly via emotional exhaustion to a statistically significant level (β=−0.095, Bootstrap 95% CI [−0.152, −0.046], *p*=.001), accounting for 34.8% of the total effect. The total effect of emotional support on turnover intention (direct + indirect) was also shown to be statistically significant (β=−0.273, Bootstrap 95% CI [−0.364, -0.175], *p*=.002). The mediating effects are outlined in Tables 4 and 5, and the mediating effect model is shown in Figure 1.

**Table 4:** Model Fit and Path Analysis of Emotional Support, Emotional Exhaustion, and Turnover Intention

Model Fit Indices					
χ²(*df*)	CFI	TLI	AIC	RMSEA [95% CI]	SRMR
0.000(0)	1.000	1.000	18.000	—	—
Path Analysis Results					
Path		***SE***	β	CR	***p***
Emotional support → emotional exhaustion		0.047	−.213	−4.495	<.001
Emotional exhaustion → turnover intention		0.043	.446	10.491	<.001
Emotional support → turnover intention		0.043	−.178	−4.179	<.001

*Note.* AIC = Akaike Information Criterion; CFI = Comparative Fit Index; CR = critical ratio; RMSEA = Root Mean Square Error of Approximation; *SE* = standard error; SRMR = Standardized Root Mean Square Residual; TLI = Tucker–Lewis Index.

**Table 5:** Mediation Analysis and Bootstrap Validation of Emotional Support, Emotional Exhaustion, and Turnover Intention

Type of Effect	Path	β	Bootstrap CI (95%)	*p*
Direct effect	Emotional support → turnover intention	−.178	[−0.255, −0.073]	<.001
	Emotional exhaustion → turnover intention	.446	[0.364, 0.551]	<.001
Indirect effect	Emotional support → turnover intention	−.095	[−0.152, −0.046]	.001
Total effect	Emotional support → turnover intention	−.273	[−0.364, −0.175]	.002

## Discussion

### Analysis of Turnover Intention in ED Nurses

The ED nurses in this study earned an overall mean score for turnover intention of 14.59 (*SD*=3.69), with the highest percentage in turnover intention III (69.5%). This indicates the participants were fairly likely to seek external job opportunities, which is consistent with the findings of Ma et al. ( 2022). As core members of the resuscitation team, ED nurses are typically required to possess a comprehensive range of nursing skills that allow them to adapt quickly to new work environments, even after transfer to other departments. The adaptability and transferability of these skills not only enhance their competitiveness in the job market but also contribute to an increased likelihood of pursuing positions in other health care organizations, further exacerbating staffing shortages in ED settings (Ganesh, 2023). Also, the 70.3% of participants who indicated having a high or very high level of turnover intention underscores the precarious situation currently faced by ED nursing teams. This high turnover intention reflects both the challenging nature of the ED work environment and the considerable strain placed on health care organizations attempting to maintain stable nursing teams. The high likelihood that ED nurses will leave their jobs after finding a more suitable opportunity necessitates urgent and appropriate interventions by management.

### Predictors of Turnover Intention in ED Nurses

#### Impact of Self-perceived Economic Status on Turnover Intention

Financial satisfaction (as reflected in self-perceived economic status) was identified as a key factor of influence on intent to leave, particularly in those “very dissatisfied” with their financial situation. This finding agrees with that of Jiang et al. (2017), which highlights the influence of wage level and other job resources on job satisfaction and subsequent intention to leave. In response to these findings, implementing tiered salary increases based on experience and role seniority can serve as a powerful incentive for long-term retention. Also, performance-related bonuses may be introduced to recognize and reward outstanding work, directly impacting economic satisfaction and reducing turnover intentions (Bowers et al., 2022). Furthermore, financial strategies should incorporate transparent communication about salary structures and benefits to foster trust and reduce uncertainties regarding compensation. Transparency is critical to ensuring employees feel valued and fairly compensated, which is key to building organizational commitment. Hospitals may also consider offering educational subsidies or housing benefits to alleviate financial burdens, particularly for younger nurses and those with families. These targeted incentives would not only address immediate financial concerns but also signal institutional support for nurses’ long-term professional and personal growth.

#### Impact of Physical Condition on Turnover Intention

Physical condition was found to significantly influence turnover intention, with participants in poor health most likely to leave, followed by those in relatively poor health. This finding aligns with a recent study conducted in Germany (Rahnfeld et al., 2023), which highlighted the critical role of personal health in determining whether nurses remain or leave their profession. The intense workload in EDs can make it difficult for nurses with poor health to cope, prompting them to seek other career opportunities to avoid long-term health risks associated with clinical nursing (Babapour et al., 2022). In the context of CART, physical health is a vital personal resource, the depletion of which, often exacerbated by adverse workplace conditions, directly contributes to increased turnover intention. To address this challenge, hospitals should develop targeted health wellness programs that include regular health screenings and ergonomic assessments to prevent work-related injuries. Proactively preventing work-related injuries is essential for preserving nurses’ physical well-being and maintaining workforce stability. Nursing leaders should also implement strategic work shift adjustments to reduce the burden of working long-hour shifts consecutively, which is often a significant contributor to physical and mental fatigue. Providing nurses with sufficient opportunities to recover between shifts is crucial to ensuring sustainable work performance. Moreover, hospitals may establish structured physical fitness programs to enhance physical resilience in their nurses. Encouraging participation in activities such as yoga, Pilates, and light exercise can help alleviate stress, improve overall health, and reduce the likelihood of work-related injuries. These initiatives can also foster a culture of health and well-being, demonstrating institutional commitment to supporting the workforce.

#### Impact of Independently Choosing a Nursing Career on Turnover Intention

Participants who had voluntarily chosen nursing demonstrated less intention to leave than their non-voluntary counterparts. This may be due to differences in psychological alignment with the nursing role and the presence (or absence) of intrinsic motivation, both of which significantly impact job satisfaction and emotional engagement with the profession. For instance, a study by Tolksdorf found nurses who were highly satisfied with their jobs and deeply identified with the nursing profession to be less likely to leave the profession (Tolksdorf et al., 2022). These findings suggest that voluntary career choice fosters a deeper sense of psychological compatibility with the profession, reducing turnover intention. Moreover, nurses who proactively chose a nursing career independently often have clear career goals, leading to higher job satisfaction as they achieve or work toward these aspirations (Lehtonen et al., 2021). Furthermore, positive perceptions of career advancement opportunities significantly reduce turnover rates, with nurses who perceive ample advancement opportunities and feel supported in their career goals being more inclined to remain in the profession (Ike et al., 2023). These findings underscore the importance of personal career choices in fostering long-term job commitment and highlight the critical role of health care organizations in supporting career development. Hospital administrators should consider offering professional growth and career development opportunities such as continuing education programs and skills enhancement workshops (Sinha, 2020) to help nurses clarify their career trajectories and enhance their sense of accomplishment. By fostering an environment that supports career development, hospitals can improve job satisfaction and reduce turnover intentions among nursing staff. Facilitating internal career mobility through mentorship programs can also empower nurses to pursue specializations that align with their interests, thus enhancing job satisfaction and retention. Furthermore, providing structured career development frameworks, including competency-based promotions and leadership opportunities, can strengthen the commitment of nurses who proactively chose a career in nursing. Such frameworks not only motivate these individuals but also foster a culture of professional growth that benefits the entire nursing workforce.

#### Impact of Emotional Support on Turnover Intention

In this study, the mean emotional support score of the participants was 18.81 (*SD*=2.74), indicating a moderate level of emotional support from their coworkers. Multiple stepwise regression analysis confirmed emotional support as significantly influencing turnover intention, with higher levels of perceived emotional support associated with lower turnover intention. This underscores the critical role of emotional support in mitigating the stress of ED work environments and enhancing nurse retention. Given the inherently high-pressure and fast-paced nature of ED work, nurses often face substantial emotional and psychological stress (Frenzel et al., 2023). In this context, emotional support from coworkers serves as a crucial resource, providing psychological comfort, reducing burnout, and strengthening team cohesion, which collectively contribute to lower turnover intention. Hospital administrators and team leaders should recognize the importance of optimizing the emotional support network. Management strategies should include structured initiatives aimed at enhancing emotional support, such as organizing peer support groups, regular mental health workshops, and debriefing sessions following high-stress events. Strengthening the emotional connection among nursing team members through these activities can help improve job satisfaction and promote retention (Bowers et al., 2022).

#### Impact of Emotional Exhaustion on Turnover Intention

In this study, the mean emotional exhaustion score of the participants was 12.06 (*SD*=4.61), reflecting a high prevalence of emotional exhaustion. Multiple stepwise regression analysis further confirmed emotional exhaustion as a significant predictor of turnover intention, with higher levels of emotional exhaustion associated with a higher propensity to leave. This finding underscores the crucial role of management in reducing job stress and preventing burnout. Emotional exhaustion not only impacts mental health in nurses but also decreases job satisfaction, further triggering turnover intention (Ding & Wu, 2023). Therefore, health care organizations should provide regular mental health assessments and counseling services to support nurses in coping with the emotional challenges of high-pressure environments. This may include establishing easily accessible psychological support hotlines and arranging structured group or individual counseling sessions, which provide a safe space for nurses to address their stress and emotional challenges (Wubetie et al., 2020). Regular mental health assessments and debriefing sessions may be used to effectively manage and replenish emotional resources, further reducing turnover intentions among nursing staff. In addition, health care organizations may consider integrating traditional Chinese wellness practices such as Tai Chi and Qigong into regular wellness programs (Liu et al., 2020b). These practices, known for their stress-reducing benefits, can be particularly effective in settings characterized by high levels of emotional exhaustion, providing nurses with tools to enhance emotional resilience and psychological well-being. Management should also address the systemic causes of emotional exhaustion, such as unclear job expectations or insufficient workplace support, by implementing workload redistribution and creating transparent communication channels. Clear and realistic job expectations can alleviate stress, while equitable redistribution of workloads ensures no single nurse bears an excessive burden.

#### Impact of Mental Health on Turnover Intention

The participants in this study held generally negative attitudes toward their mental health, with a mean score of 3.42 (*SD*=0.814), which is consistent with previous research (Charzyńska et al., 2023) and highlights the psychosocial work pressures faced by ED nurses. These pressures are often exacerbated by high job demands, limited autonomy in the work environment, restricted access to social support, and insufficient opportunities to engage in physical activities (Sarafis et al., 2016; Thapa et al., 2022). To address these challenges, health care organizations should implement comprehensive mental health support systems tailored to the unique needs of ED nurses. For example, the introduction of anonymous mental health support services can provide a confidential platform for nurses to seek assistance without fear of stigma. Incorporating mental health education into ongoing professional development programs can also enhance nurses’ understanding of mental health management, equipping them with practical tools to cope with stress and improve their resilience. Creating a workplace culture that normalizes seeking mental health support is essential in reducing stigma and promoting early intervention. However, this necessitates the active effort of hospital management to promote open discussions about mental health, train team leaders to recognize the early signs of psychological distress and provide readily available support resources. By addressing mental health proactively and fostering a culture of care, health care organizations can significantly reduce turnover intentions and enhance overall well-being among their nursing staff.

### Mediating Role of Emotional Support

Emotional support both reduces turnover intention among ED nurses directly and impacts turnover intention indirectly by alleviating emotional exhaustion. This dual role highlights the critical importance of fostering emotional support systems to improve retention and enhance job satisfaction. Specifically, the inclusion of emotional exhaustion as a mediator in the model significantly reduced the direct effect of emotional support on turnover intention, underscoring the mediating role of emotional exhaustion. To capitalize on the protective role of emotional support, health care organizations should prioritize the development of robust support systems tailored to the unique challenges of ED nursing. For instance, establishing structured peer support networks such as nurse-led discussion groups can create safe spaces for sharing experiences and receiving guidance. These networks can foster a sense of community and solidarity, which is essential for reducing stress and burnout. Furthermore, recognizing and rewarding emotional contributions within the team through, for example, peer acknowledgment programs can further bolster emotional support.

Leadership training on emotional intelligence is another essential intervention. Equipping managers with the skills necessary to recognize the early signs of emotional exhaustion and provide timely emotional support can significantly enhance team cohesion and morale. Such interventions also empower leaders to create an empathetic and understanding work environment, further promoting nurse retention. Creating a workplace culture that values emotional support should be a top priority for health care organizations. By fostering a workplace culture that values emotional support and prioritizes the well-being of nurses, health care organizations can mitigate turnover intentions, reduce emotional exhaustion, and build resilient and satisfied nursing teams.

### Conclusions

Multiple factors affecting turnover intention in nurses working in hospital EDs in China, including financial satisfaction, physical health status, autonomous career choice, mental health status, emotional exhaustion, and emotional support, were comprehensively examined in this study. The findings highlight the interactions among these factors and their impact on turnover intention in a high-pressure work environment. The findings suggest that due to the high job demands and comprehensive nursing skills required in EDs, nurses are more likely to feel emotionally depleted and seek external career opportunities. Emotional exhaustion emerged as a key driver of turnover intention, while adequate emotional support significantly mitigated this tendency, highlighting the crucial mediating role of emotional support in reducing employee turnover. The high turnover rates currently observed among ED nurses in China highlight critical challenges in nursing human resource management and underscore the urgent need for supportive and sustainable work environments. Hospital administrators are encouraged to enhance working conditions by implementing measures such as regular mental health assessments, providing comprehensive mental health support services, and fostering stronger team cohesion and emotional connectivity. These interventions are not merely supportive measures but are also strategic imperatives that can substantially reduce emotional exhaustion and turnover rates. Moreover, targeted interventions designed to boost emotional support within nursing teams can effectively mitigate the negative consequences of emotional exhaustion. In prioritizing these areas, management can significantly improve the stability and cohesion of nursing teams, thereby enhancing the quality of patient care delivered. Adopting these management strategies offers dual benefits: improving the quality of nurses’ professional lives while positively impacting the overall operational and service quality of health care organizations. This approach not only aligns with the goals of reducing turnover but also will contribute to a more resilient health care system in China.

### Limitations

There were several limitations to this study. First, the sample was drawn only from general hospitals in one region in China. Thus, extrapolation of the results may be limited and may not fully represent the turnover intentions of all ED nurses. Second, the cross-sectional design used in this study only described correlations and did not allow for the inference of causal relationships between variables. To overcome these limitations, future studies should consider adopting a longitudinal research design, which would help track and analyze changes in turnover intention over time and their potential drivers. Moreover, using probability sampling methods across various regions is recommended to enhance the representativeness and generalizability of the study results. By collecting data at multiple points in time, future research can more accurately capture the trends and dynamics of nurses’ intention to leave their jobs.
